# Long‐Term Outcomes of Radiofrequency Atrioventricular Node Ablation in a Real‐World Population

**DOI:** 10.1111/pace.70196

**Published:** 2026-03-10

**Authors:** Vanessa Sciacca, Nora‐Kristin Brandt, Thomas Fink, Denise Guckel, Maximilian Mörsdorf, Yuri Bocchini, Martin Braun, Moneeb Khalaph, Karen Harutyunyan, Nadica Trajkovska, Philipp Lucas, Thomas Eitz, Maxim Didenko, Philipp Sommer, Christian Sohns

**Affiliations:** ^1^ Clinic For Electrophysiology Herz‐ und Diabeteszentrum NRW Ruhr‐Universität Bochum Bad Oeynhausen Germany

## Abstract

**Background:**

While interventional strategies have expanded the options for long term rhythm restoration, rate control continues to play a pivotal role in the treatment of atrial arrhythmias; however, pharmacologic strategies alone often fail in achieving effective rate control.

**Aims:**

To examine long‐term outcomes in patients who have undergone atrioventricular node ablation (AVNA) for the management of symptomatic atrial arrhythmias.

**Methods:**

This observational Study Assessed Patients Who Underwent AVNA For Rate Control of Atrial Fibrillation (AF) Or Atrial Tachycardia (AT) Between April 2014 and February 2022. Clinical data, along with follow‐up information including cardiac device interrogation were analyzed. A composite safety endpoint, Encompassing Heart Failure (HF) Rehospitalization, lead revision, device infection, or upgrade for cardiac resynchronization therapy (CRT), was evaluated. Additionally, structured patient interviews were conducted to assess quality of life outcomes.

**Results:**

192 patients (76 females (39.6%), mean age 73.7 ± 10 years) were included into the study. Patients suffered from paroxysmal AF in 10 cases (5.2%), persistent AF in 138 cases (71.9%) and AT in 44 cases (22.9%). Acute AVNA was successful in all patients. Two pseudoaneurysms at the femoral puncture site occurred as the only periprocedural complications. Mean follow‐up duration was 907.0 ± 609.7 days. Persistent complete AV block was present in 191 patients (99.5%) during follow‐up. The composite safety endpoint occurred in 58 (30.2%) patients. Quality of life significantly improved in most patients with a relevant regression in EHRA and NYHA scores.

**Conclusion:**

AVNA is effective and safe in an all‐comer patient population with high success rates in terms of rate control, QOL improvement and a favorable safety profile during long‐term observation.

## 1 Introduction

Atrial fibrillation (AF) and atrial tachycardia (AT) are common cardiac arrhythmias associated with elevated morbidity and mortality and significantly impaired quality of life. Restoration and long‐term maintenance of sinus rhythm remains a major clinical challenge. Despite considerable progress in interventional rhythm control, a relevant subset of patients fails to achieve sustained rhythm stability. Therefore, rate‐controlling strategies continue to play a pivotal role in the management of atrial arrhythmias.

Guidelines endorse rate control strategies in AF with a target resting heart rate below 110 bpm [1]. AVNA combined with pacemaker implantation has been increasingly utilized in patients resistant to pharmacologic rate control or with recurrent atrial tachyarrhythmias [1]. Various studies report that AVNA can improve quality of life, reduce symptoms, reduce hospital admissions, and reduce antiarrhythmic medication [2, 3]. However, potential complications have limited its widespread usage. These concerns include pacing‐induced cardiomyopathy, as well as other device‐related issues such as lead dislodgement, device infection or dysfunction [4, 5, 6, 7].

## 2 Methods

### 2.1 Study Population

This observational study included consecutive patients who underwent atrioventricular node ablation (AVNA) between April 2014 and February 2022 for AT or AF refractory to pharmacologic rate control, with or without prior rhythm control attempts. The study was approved by the local ethic committee. All patients gave written informed consent to participate in this study. The study did not receive funding.

### 2.2 Primary Endpoint

A composite safety endpoint was evaluated, comprising rehospitalization for heart failure (HF), lead revision, device infection, or upgrade to cardiac resynchronization therapy (CRT) due to suspected pacing‐induced cardiomyopathy. This composite endpoint was chosen to reflect the overall long‐term burden of the ablate‐and‐pace strategy in a real‐world setting, capturing both clinically relevant outcomes and device‐related complications directly attributable to permanent pacing.

### 2.3 Secondary Endpoints

Secondary endpoints were recovered AV conduction after AVNA and patient reported outcomes including QOL.

### 2.4 Pacemaker Implantation

All patients underwent pacemaker implantation prior to AVNA. Pacemaker implantation was either performed for the purpose of AVNA or had been carried out previously due to symptomatic bradycardia. The selection of the pacemaker manufacturer, pulse generator model, pacing mode, and lead configuration was determined at the discretion of the operating physician in accordance to current guidelines [2]. For patients receiving a permanent pacemaker specifically for AVNA, a CRT device was implanted if left ventricular ejection fraction (LV‐EF) was below 50% with or without previous HF hospitalization. No mandatory waiting period was enforced between pacemaker implantation and AVNA. Among patients with a prior cardiac device implanted for reasons other than AVNA, a baseline LVEF < 50%, and no existing CRT system, we performed AVNA initially and conducted intensive follow‐up, proceeding to CRT upgrade when warranted. Device type at the time of AVNA (pacemaker, ICD, CRT‐P, CRT‐D) is reported in Table 1. A separate outcome analysis by pacing mode (e.g., VVI vs. DDD) was not performed, since pacing modes were frequently adapted during follow‐up depending on atrial rhythm status and clinical requirements.

**TABLE 1:** Baseline data.

Patients, *n*	192
Gender, *n* (%)	
Male	116 (60.4)
Female	76 (39.6)
Age	73.7 ± 10
BMI [kg/m^2^]	28.5 ± 5.5
Structural heart disease, *n* (%)	
CAD	81 (42.2)
NICM	50 (26.0)
LV‐EF [%]	37.4 ± 13.8
LV‐EF ≤ 35%, *n* (%)	104 (54.2)
Device implantation to AVNA (days)	959 {49.5; 2879}
Cardiac device at time of AVNA, *n* (%)	
Pacemaker	60 (31.3)
ICD	15 (7.8)
CRT‐P	22 (11.5)
CRT‐D	95 (49.5)
Leading arrhythmia before AVNA, *n* (%)	
PAF	10 (5.2)
PersAF	138 (71.9)
AT	44 (22.9)

Abbreviations: AT, atrial tachycardia; AVNA, atrioventricular node ablation; BMI, Body mass index; CAD, coronary artery disease; CRT‐D, cardiac resynchronization therapy defibrillation; CRT‐P, cardiac resynchronization therapy pacing; ICD, implantable cardioverter defibrillator; LV‐EF, left ventricular ejection fraction; NICM, non‐ischemic cardiomyopathy; PAF, paroxysmal atrial fibrillation; PersAF, persistent atrial fibrillation.

### 2.5 Ablation Approach

AVNA was conducted under deep sedation, utilizing propofol, midazolam, and fentanyl. Direct oral anticoagulants were withheld on the morning of the procedure, while patients on vitamin K antagonists continued their regimen without interruption, maintaining an INR target of 2–3. A single right‐sided femoral puncture was performed, through which an 8F sheath was introduced. The ablation catheter was advanced into the right atrium and positioned slightly caudal to a site exhibiting a large‐amplitude atrial deflection with a small His bundle potential. Ablation was executed either with conventional fluoroscopic guidance or using a 3D mapping system [4, 5]. Given the anatomical complexity of the AV junction and variability in the inferior nodal extensions, the use of electroanatomic mapping systems can facilitate precise identification of the compact node and fast‐pathway inputs [8, 9]. The procedure was carried out using an irrigated‐tip ablation catheter (Navistar Thermocool, Biosense Webster; Flexibility, Abbott) at power settings of 30–40 W, typically for 60–120 s per application. If complete atrioventricular block was not immediately induced, catheter repositioning and further application of radiofrequency energy were performed. The endpoint of the procedure was the induction of complete heart block, preferentially at the most proximal part of the His bundle to preserve an escape rhythm. A 15‐minute waiting period following the induction of complete heart block was held in all patients. In cases of recurrent atrioventricular conduction, repeat ablation was performed. After sheath removal, a femoral figure‐of‐eight suture and a pressure bandage were applied to prevent bleeding. Pressure bandages were removed 6 h post‐procedure, and femoral sutures were removed the following morning. Transthoracic echocardiography was conducted immediately after the procedure and the following morning to rule out pericardial effusion. For patients on oral anticoagulation, medication was resumed six hours post‐ablation.

### 2.6 Periprocedural Device Programming

Prior to AVNA, device interrogation was performed to verify proper functioning of the device and leads. All cardiac devices were then programmed to a ventricular pacing mode at a rate of 30 beats per minute (VVI 30/min) at the beginning of the ablation procedure. After the procedural endpoint was achieved, the device was again interrogated to ensure correct function and subsequently programmed to DDIR/VVIR 80/min in all patients. This elevated lower rate limit was maintained for 4 weeks post‐ablation and then individually adapted based on patients’ characteristics. This standardized peri‐procedural programming strategy was applied irrespective of baseline device type.

### 2.7 Clinical Follow‐Up

Clinical follow‐up was initially scheduled at 6 and 12 months, with subsequent visits every 6 months thereafter, either at our outpatient clinic or with referring physicians. During these follow‐up visits, patients underwent routine transthoracic echocardiography, electrocardiography (ECG), and device interrogation. The device interrogation primarily focused on assessing lead and device functionality, as well as confirming the persistence of complete heart block. The clinical impact of arrhythmia symptoms was evaluated using the modified EHRA score [1]. Additionally, a structured quality‐of‐life questionnaire was completed by each patient either during the clinical visit or via a telephone interview, assessing changes in NYHA classification and patients’ statements on satisfaction with AVNA.

### 2.8 Statistical Analysis

Continuous variables were presented as mean ± standard deviation or standard error of the mean for normally distributed data, and as median with interquartile range for non‐normally distributed data. Categorical variables were expressed as frequencies (%). Comparisons of continuous variables between two groups were conducted using the Student's *t*‐test, while analysis of multiple groups was performed using ANOVA. Survival analysis was carried out employing the Kaplan‐Meier method with group comparisons utilizing a log‐rank test. Multivariable regression analyses were performed to identify independent predictors of the composite endpoint, HF rehospitalization, and all‐cause mortality, including clinically relevant baseline covariates. All reported *P*‐values are two‐sided, with values < 0.05 considered statistically significant. Statistical analyses were performed using SPSS version 25 (IBM Corporation, Armonk, NY, USA).

## 3 Results

### 3.1 Baseline Data

The study group consisted of 192 patients (116 males, 60.4%) with a mean age of 73.7 ± 10 years. Structural heart disease was present in 131 patients (68.2%). Mean LV‐EF was 37.4% ± 13.8%. Median time between device implantation and AVNA was 959 days {49.5;2879}. The arrhythmia leading to AVNA was paroxysmal AF in 10 patients (5.2%), persistent AF in 138 patients (71.9%) and AT in 44 patients (22.9%). At the time of AVNA, 60 patients (31.3%) had a pacemaker, 15 (7.8%) an ICD, 22 (11.5%) a CRT‐P system, and 95 (49.5%) a CRT‐D system. In 163 patients (84.9%) the implanted cardiac device included an atrial lead. All patients underwent pharmacological rate control and/or antiarrhythmic drug (AAD) therapy prior to AVNA. Overall, 153 patients (79.7%) had received sequential trials of multiple AADs before the intervention. Amiodarone was prescribed in 117 patients (61.0%), flecainide or propafenone in 31 (16.1%), and sotalol in 14 (7.3%). Concomitant rate‐controlling therapy with digitalis and/or beta‐blockers was used in 190 patients (99.0%). Table 1 summarizes further baseline data.

### 3.2 Procedural Data

Mean procedural duration for AVNA was 33.5 ± 24.3 min and a mean fluoroscopy time of 5.3 ± 2.4 min. Most procedures were performed with implementation of a 3D‐mapping system (94.8%). In two patients (1.0%) a pseudoaneurysm at the femoral puncture site occurred. Both cases could be managed with manual compression. No other periprocedural complications occurred. Table 2 summarizes procedural data.

**TABLE 2:** Procedural data.

Procedural duration [min]	33.5 ± 24.3
Fluoroscopy time [min]	5.3 ± 2.4
Procedural complications, *n* (%)	2 (1)
Lead dislodgement	0 (0.0)
Pericardial effusion	0 (0.0)
Pseudoaneurysm at puncture site	2 (1.0)

### 3.3 Follow‐Up

**FIGURE 1:**
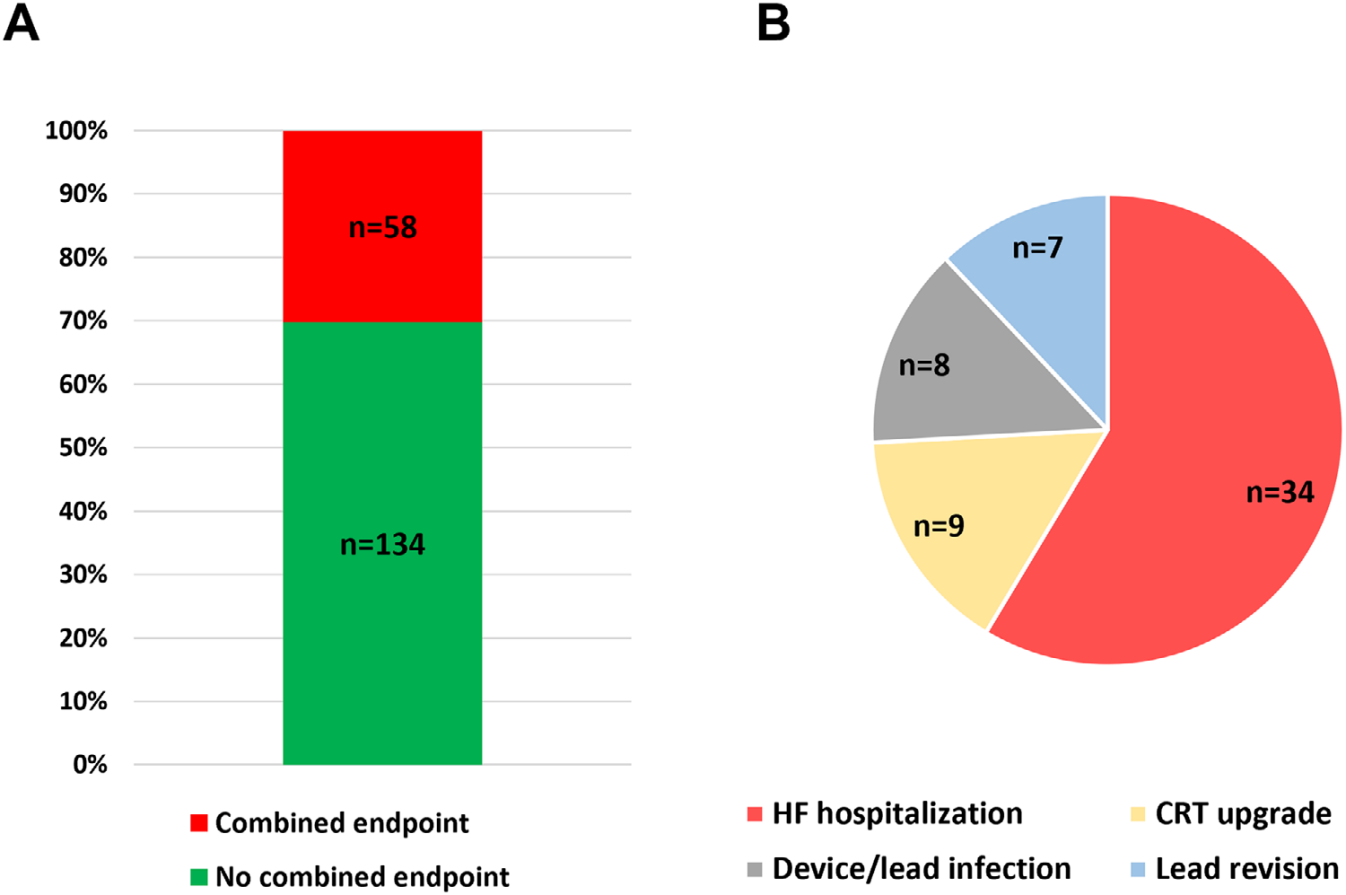
Incidence and composition of the composite safety endpoint during follow‐up. (A) Cumulative incidence of the composite safety endpoint during follow‐up after atrioventricular node ablation (AVNA). (B) distribution of individual components contributing to the composite endpoint, including heart failure (HF) rehospitalization, device infection, lead revision, and upgrade to cardiac resynchronization therapy (CRT). HF rehospitalization represents the predominant clinical component of the composite endpoint. [Colour figure can be viewed at wileyonlinelibrary.com]

**FIGURE 2:**
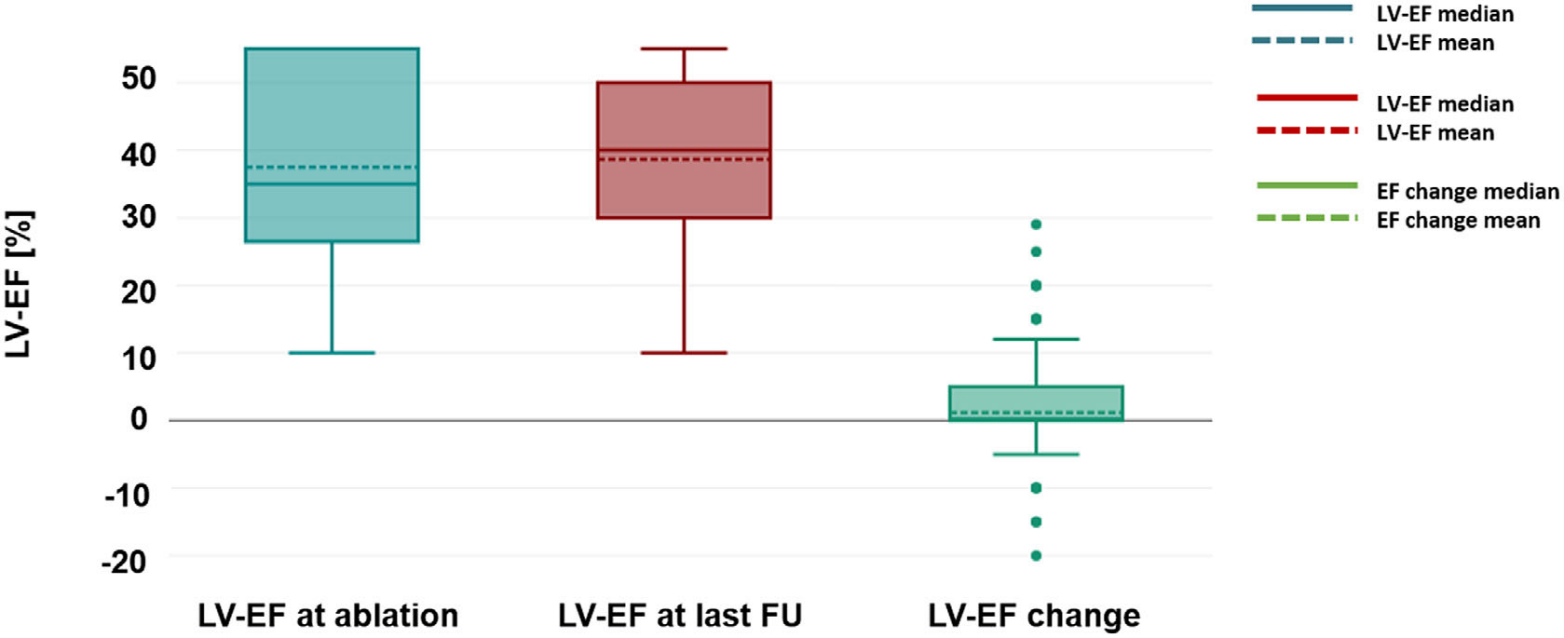
Changes in left ventricular ejection fraction following atrioventricular node ablation. Left ventricular ejection fraction (LV‐EF) at baseline and at last follow‐up after AV node ablation (AVNA). Individual patient trajectories are shown, together with mean and median changes in LV‐EF over time. Patients undergoing CRT upgrade are included in the analysis according to their last available echocardiographic assessment. [Colour figure can be viewed at wileyonlinelibrary.com]

**FIGURE 3:**
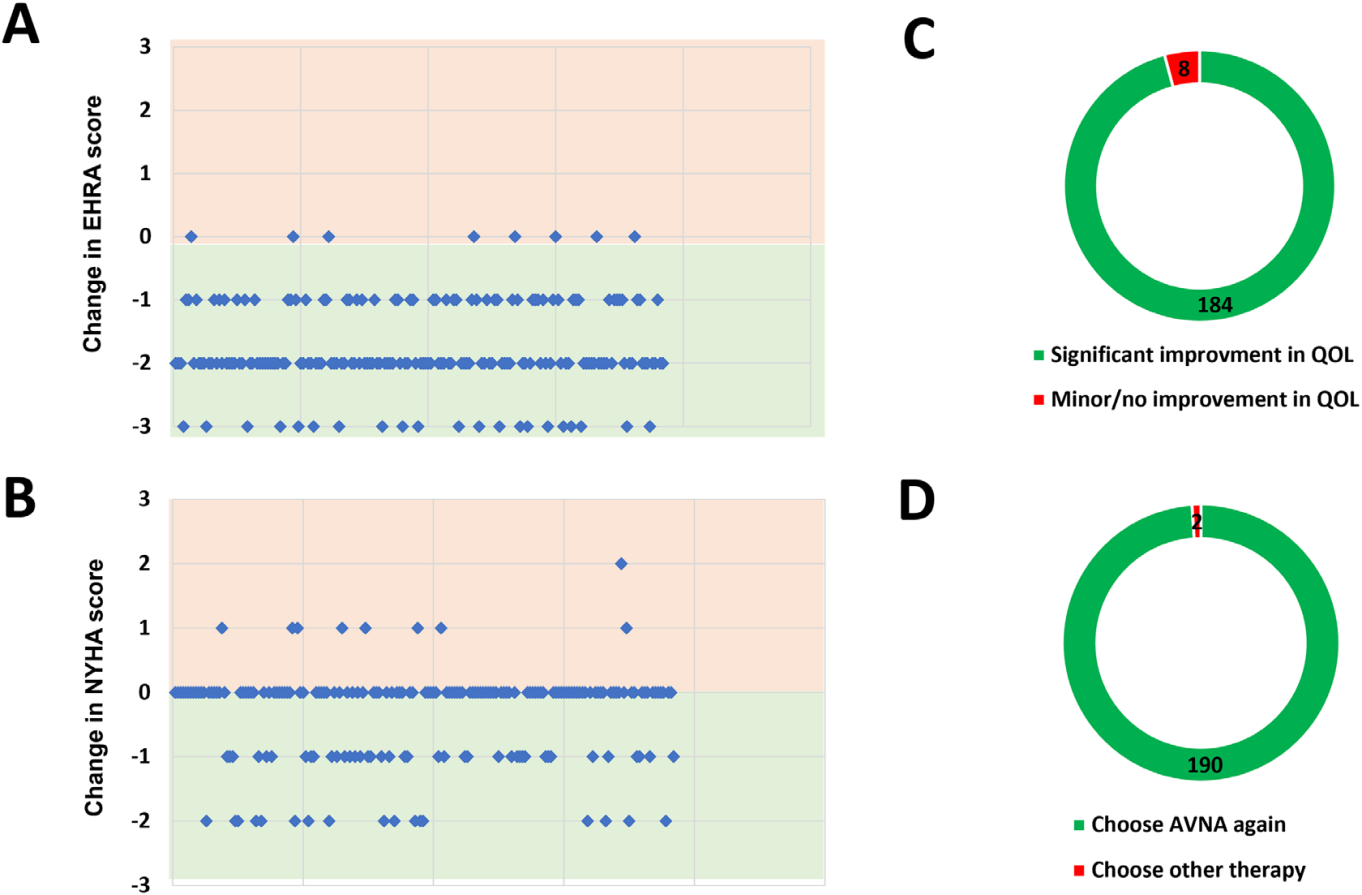
Patient‐reported outcomes and quality‐of‐life measures following atrioventricular node ablation. (A) change in EHRA symptom class before and after AVNA. (B) change in NYHA functional class following AV node ablation (AVNA). (C) patient‐reported overall satisfaction with AVNA. (D) willingness to undergo AVNA again for arrhythmia management. Collectively, these measures reflect patient‐centered improvements in quality of life (QOL), functional status, and treatment satisfaction. [Colour figure can be viewed at wileyonlinelibrary.com]

Mean follow‐up duration was 907.0 ± 609.7 days. Persistent complete heart block throughout the entire follow‐up period was observed in all but one patient (99.5%). Repeat AVNA was performed in this patient. No rapid AV conduction of AF or AT was documented during follow‐up in patients with persistent complete AV block. An intrinsic escape rhythm was observed in 32 patients (16.7%) during follow up. At the time of last clinical follow‐up adequate rate control was achieved in all patients. AAD therapy for AF/AT was discontinued in all patients after AVNA. During follow‐up, AAD therapy was re‐initiated in 16 patients receiving amiodarone and in 4 patients receiving sotalol. Notably, in all cases, antiarrhythmic therapy post AVNA was prescribed exclusively for the management of ventricular arrhythmias. The composite safety endpoint occurred in 58 patients (30.2%, Figure 1A). The composite safety endpoint was met due to HF rehospitalization in the majority of patients (34 patients (17.7%), Figure 1b). A CRT upgrade was performed in 9 out of 75 patients (12%) who had a conventional pacemaker or ICD system at the time of AVNA. Mean LV‐EF in patients undergoing CRT‐upgrade was 45% ± 4.1%. No patient with a baseline LV‐EF of > 50% at the time of AVNA required a CRT‐upgrade during follow‐up. Device infections were observed in 8 patients (4.2%) and lead revisions for other reasons than infection in 7 patients (3.7%). Figure 2 depicts LV‐EF at the time of last follow‐up in relation to baseline and presents the mean and median changes in LV‐EF following AVNA. Sustained ventricular arrhythmias were observed in 27 (14.1%) patients, however, none of these occurred within the first 12 weeks after AVNA. Left ventricular assist device implantation or heart transplantation was observed in 9 (4.7%) patients. A total of 41 patients died during the follow‐up period (Table 3). Most patients reported a relevant reduction of arrhythmia and HF symptoms after AVNA (Figure 3A, B). A clinically significant enhancement in quality of life after AVNA was noted by 184 patients (95.8%), while 190 patients (99.0%) expressed willingness to undergo AVNA again for arrhythmia management (Figure 3C, D).

**TABLE 3:** Follow‐up data.

Duration of FU [days]	907.0 ± 609.7
Persistent complete heart block, *n* (%)	191 (99.5)
HF rehospitalization after AVNA, *n* (%)	34 (17.7)
CRT upgrade, *n* (%)	9 (12)
Device infection, *n* (%)	8 (4.2)
Lead revision, *n* (%)	7 (3.6)
Documentation of ventricular arrhythmia, *n* (%)	27 (14.1)
LVAD/HTx during FU, *n* (%)	9 (4.7)
Death during FU, *n* (%)	41 (21.4)

Abbreviations: AVNA, atrioventricular node ablation; CRT, cardiac resynchronization therapy; FU, follow‐up; HF, heart failure; HTx, heart transplant; LVAD, left ventricular assist device.

**FIGURE 4:**
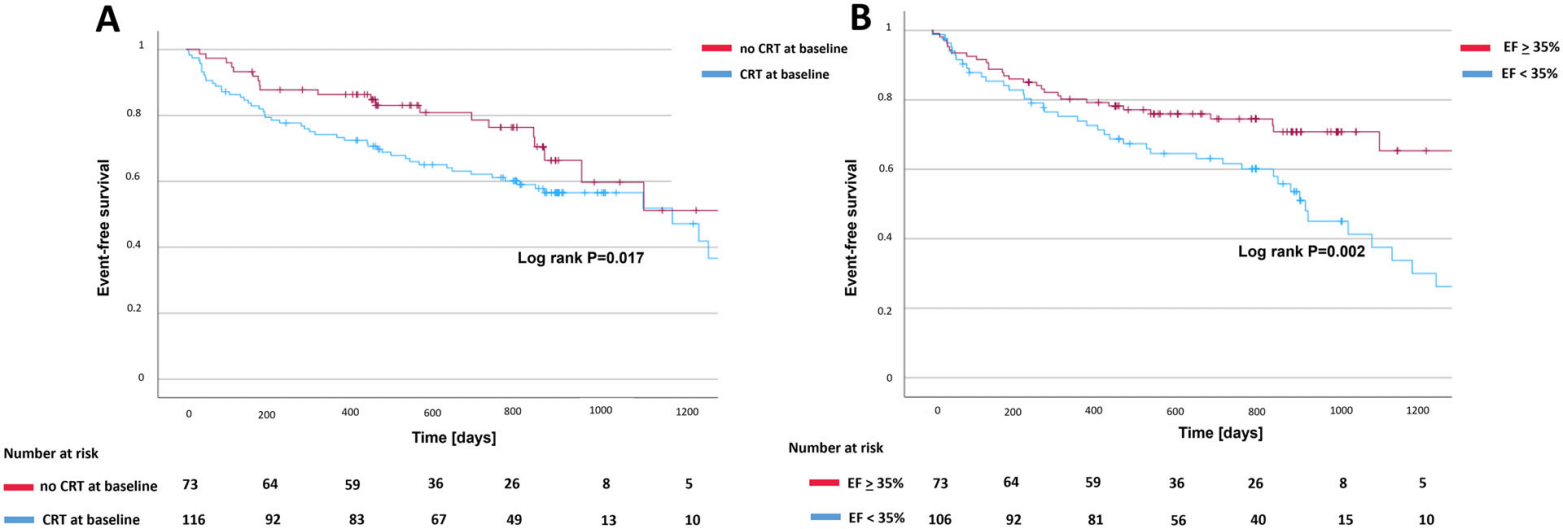
Survival analyses stratified by baseline device type and left ventricular function. (A) Kaplan–Meier curves illustrating freedom from the composite safety endpoint stratified by device type at the time of AVNA (CRT vs. conventional pacemaker/ICD). (B) Kaplan–Meier curves stratified by baseline left ventricular ejection fraction (LV‐EF < 35% vs. ≥ 35%). Patients with reduced LV‐EF and those with pre‐existing CRT systems exhibited a significantly higher incidence of the composite endpoint during follow‐up. [Colour figure can be viewed at wileyonlinelibrary.com]

Survival analysis demonstrated a significantly higher incidence of the primary composite endpoint among patients who received CRT prior to AVNA (log‐rank *p* = 0.017) compared with those with conventional pacemaker systems (Figure 4A). In addition, patients with a baseline LV‐EF < 35% showed a significantly higher event rate than those with LV‐EF ≥ 35% (log‐rank *p* = 0.002, Figure 4B). In multivariable regression analyses adjusting for age, sex, baseline left ventricular ejection fraction (LV‐EF), and cardiomyopathy subtype, reduced LV‐EF and the presence of non‐ischemic cardiomyopathy (NICM) were identified as independent predictors of the composite safety endpoint (Table 4). Similarly, lower baseline LV‐EF was independently associated with HF rehospitalization. With respect to all‐cause mortality, NICM emerged as an independent predictor, whereas LV‐EF, age, and sex were not significantly associated with mortality in the adjusted model.

**TABLE 4:** Multivariable regression analyses for clinical outcomes.

	Coef B	Standard error	*z*	*p*	Odds Ratio	95% COI
**Combined endpoint**						
**Age**	0	0.02	0.07	0.94	1	0.97–1.03
**Male sex**	−0.13	0.41	0.32	0.75	0.88	0.39–1.96
**LV‐EF [%]**	−0.04	0.01	2.54	**0.01**	0.96	0.93–0.99
**NICM**	0.94	0.36	2.58	**0.01**	2.55	1.25–5.18
**All‐cause death**						
Age	0.04	0.02	1.90	0.06	1.04	1.00–1.09
Male sex	0.60	0.48	1.25	0.21	1.82	0.71–4.62
LV‐EF [%]	−0.05	0.02	3.02	**0.003**	0.95	0.91–0.98
NICM	0.09	0.41	0.22	0.82	1.10	0.49–2.44
**HF‐rehospitalization**						
Age	0.02	0.02	0.86	0.39	1.02	0.98–1.06
Male sex	0.68	0.50	1.36	0.17	1.97	0.74–5.22
LV‐EF [%]	−0.01	0.02	0.82	0.41	0.99	0.95–1.02
NICM	0.83	0.42	2.00	0.05	2.30	1.02–5.19

Abbreviations: COI, confidence interval; LV‐EF, left ventricular ejection fraction; NICM, non‐ischemic cardiomyopathy.

## 4 Discussion

Sustained rhythm control fails in a significant proportion of patients with AF or AT despite advances in interventional therapy. When rhythm control cannot be achieved, rate control becomes a reasonable approach. Pharmacologic rate control often remains insufficient [2]. However, a major concern associated with AVNA is pacemaker‐induced cardiomyopathy, which may necessitate an upgrade to CRT or conduction system pacing (CSP) [6, 7, 10]. Further concerns include pacemaker‐related issues, such as infection and device dysfunction requiring surgical revision, as well as procedural risks like vascular access complications and lead dislodgement during AVNA [8, 9, 11, 12, 13]. The present study analyzes long‐term outcomes of AVNA in a real‐world all‐comer patient population. The main findings of the present study are:

1. AVNA was highly effective With a very low rate of recurrent AV conduction,
2. AVNA was associated With low acute and moderate long‐term complication rates,
3. AVNA was associated with a relevant reduction of arrhythmia symptoms and significant improvement in quality of life.

### 4.1 Atrioventricular Conduction Block Durability and Safety of AVNA

A large body of data exists on patients undergoing AVNA with various types of cardiac devices. Despite intensively studied clinical outcomes of patients undergoing pacemaker implantation and AVNA, there is only very limited data on the effectiveness of AVNA in terms of acute procedure success, periprocedural complications and recurrent AV conduction during follow‐up.

We have observed very low acute complication rates for catheter‐based AVNA. In our cohort, 2 patients (1%) experienced conservatively manageable complications at the femoral puncture site. No periprocedural cardiac effusion, relevant bleeding or pacemaker lead dislodgement occurred. In a smaller retrospective study, lead dislodgement due to AVNA occurred in 1.5% of the patients [8]. The APAF‐CRT Mortality trial documented lead dislodgement in 4.8% in the treatment group [4]. However, it remains unclear whether lead dislodgement was directly caused by AVNA in this study [4]. We observed acute procedural success with complete heart block at the end of the procedure in all patients. None of the procedures performed required left atrial ablation. In a retrospective case control series by Wang and colleagues, acute AVNA could not be achieved in 1.2% of the patients [14]. Long‐term rates of persistent AV block have not been reported in this cohort [14]. In a prospective, multicenter study involving a relatively large cohort of 375 patients undergoing AVNA, a recurrence rate of atrioventricular conduction of 5.7% was reported. During the long follow‐up period we have observed 1 case (0.5%) of recurrent AV conduction. The relatively low rate of recurrent AV conduction may be attributable to the high proportion of procedures performed with three‐dimensional electroanatomic mapping in our cohort [5, 8, 9]. Our findings underscore the high acute and long‐term efficacy of rate control achieved with AVNA, along with low rates of procedure related complications.

### 4.2 Device Related Adverse Outcomes Following AVNA

A major concern regarding AVNA is the risk of long‐term device‐related complications, including infection, lead dysfunction requiring surgical revision, and pacemaker‐induced cardiomyopathy in patients with conventional pacing systems, which may necessitate upgrades to CRT or CSP. In recent years, CSP, including His‐bundle pacing and left bundle branch area pacing, has gained increasing attention as a physiological pacing strategy that may reduce pacing‐induced ventricular dyssynchrony and associated cardiomyopathy. Although CSP was not utilized in the present cohort, our findings demonstrate that AVNA itself provides durable rate control and substantial symptomatic improvement irrespective of pacing modality. Consequently, the favorable clinical outcomes observed in this study primarily reflect the efficacy of AVNA as a rate‐control strategy, while contemporary pacing approaches such as CSP may further optimize ventricular function and long‐term outcomes. Future prospective studies are warranted to directly compare pacing strategies following AVNA and to define the optimal pacing approach in this setting.

Infection of the generator pocket or leads can occur at the time of cardiac device implantation or at any subsequent time and varies between different types of cardiac devices. In a study of 1,065,549 patients who underwent cardiac device implantation between 2004 and 2019, infection occurred in 11,304 patients (1.0%) [11]. In a large Danish registry, the lifetime risk of device infection was reported as 1.2% for pacemakers, 1.9% for implantable cardioverter–defibrillators, 2.2% for CRT pacemakers, and 3.4% for CRT defibrillators [12]. The predefined composite safety endpoint comprising rehospitalization forHF, cardiac device infection, lead revision, or upgrade to CRT, was met in 30.2% of patients in our cohort. However, device infection contributed to this endpoint in only a small proportion of cases (8 patients, 4.2%). Given the long follow‐up duration (907.0 ± 609.7 days) and the high proportion of patients with CRT systems (117 patients, 61%) in our study, the observed infection rate is consistent with previously reported rates. The frequency of lead failure requiring surgical revision and the nature of associated complications have been evaluated in several studies [13, 15, 16]. In a cohort of 1,317 consecutive patients who underwent ICD implantation between 1993 and 2004 and were followed for a median of 6.4 years, 2.9% experienced lead malfunction necessitating revision [13]. In a Danish nationwide registry including 28,860 patients with pacemaker or CRT pacemaker leads, the incidence of any lead‐related complication was 3.6% [15]. In our cohort, lead dysfunction necessitating surgical revision was observed in 3.6% of patients, consistent with rates reported in previous studies. Right ventricular pacing‐induced cardiomyopathy develops in 6% to 25% of patients after right ventricular pacemaker implantation, necessitating an upgrade to CRT or CSP [6, 7]. Several studies have demonstrated the role of underlying His‐Purkinje conduction reserve and the anatomical variability of the AV node and its extensions in determining susceptibility to dyssynchrony [10, 17, 18, 19]. We observed CRT upgrade due to right ventricular pacing induced cardiomyopathy in 9 patients (4.7%). No patient with a baseline LV‐EF of > 50% at the time of AVNA required a CRT‐upgrade during follow‐up. Our data suggest that even in a long‐term follow up, device‐related adverse events (e.g., device infection, lead revision, CRT upgrade) occur with a comparably low incidence in a large real‐world patient population undergoing AVNA.

### 4.3 Rehospitalization Rates for HF Following AVNA

A relevant proportion of patients in our study experienced rehospitalization for HF following AVNA, consistent with previous reports. In line with these observations, multivariable regression analyses identified baseline left ventricular ejection fraction and underlying cardiomyopathy subtype as independent predictors of long‐term outcomes after AVNA. Reduced LV‐EF was independently associated with both the composite safety endpoint and HF rehospitalization, underscoring the prognostic relevance of ventricular function at the time of AVNA. In addition, non‐ischemic cardiomyopathy emerged as an independent predictor of all‐cause mortality. These findings highlight the importance of baseline myocardial substrate in determining long‐term prognosis following AVNA and support careful patient selection and individualized pacing strategies in patients with advanced ventricular dysfunction. In the APAF‐CRT study, which randomized patients to optimized CRT or right ventricular apical pacing following successful AV junction ablation, 11.9% of patients were hospitalized for HF during a median follow‐up of 20 months, despite a mean baseline LV‐EF that was moderately reduced [4]. Experimental and clinical observations suggest that the extent of escape rhythm suppression, His‐Purkinje system integrity, and nodal input patterns influence post‐ablation hemodynamics [10, 17, 18]. In our cohort, HF rehospitalization accounted for the largest share of events contributing to the composite safety endpoint. These findings underscore the prognostic role of left ventricular function at the time of AVNA and align with previously published data suggesting that patients with impaired systolic function may derive greater benefit from upfront CRT implantation or CSP to mitigate the risk of pacing‐induced ventricular dysfunction.

### 4.4 Quality of Life Improvement

Quality of life (QOL) outcomes represent a critical endpoint in patients undergoing AVNA, particularly when rhythm control strategies have failed and the goal shifts toward symptom relief and functional improvement. In our cohort, patient‐reported outcomes revealed substantial improvements in both arrhythmia‐related symptoms and HF burden, as reflected by favorable changes in EHRA and NYHA scores. Notably, 95.8% of patients reported a clinically meaningful improvement in QOL following AVNA, and 99% indicated they would opt to undergo the procedure again, suggesting high levels of patient satisfaction. These findings corroborate earlier studies demonstrating enhanced QOL, reduced hospitalizations, and increased functional status in patients undergoing AVNA in conjunction with device therapy [3, 4, 20].

### 4.5 Limitations

The study has an observational character and is therefore associated with the typical limitations regarding data quality. Moreover, quality‐of‐life assessment was limited to NYHA and EHRA scores and patient‐reported satisfaction, whereas validated multidimensional instruments were not available for systematic analysis. In addition, detailed long‐term pacing mode stratification (e.g., VVI vs. DDD) was not feasible because pacing mode was frequently modified during follow‐up according to rhythm status and clinical needs. The follow‐up period in this study is relatively long compared to previously published data. However, cardiac device related complications might be even higher with longer follow‐up durations. A larger patient cohort might have further altered complications and success rates. Moreover, our patient cohort does not comprise patients with CSP or leadless pacing; therefore, the reported outcomes reflect conventional pacing and CRT strategies and may not be directly extrapolated to contemporary CSP‐based approaches. Further larger and prospective studies are needed to explore potential benefits and risks of AVNA for rate control.

## 5 Conclusions

AVNA was associated with high success rates in terms of long‐term rate control for AF and AT in a real‐world patient population. High rates of patient satisfaction, significant QOL improvements and low rates of adverse events were observed after AVNA over an extended follow‐up period.

## Conflicts of Interest

The authors declare no conflicts of interest.

## Data Availability

The data that support the findings of this study are available from the corresponding author upon reasonable request.
